# Blunted IL17/IL22 and Pro-Inflammatory Cytokine Responses in the Genital Tract and Blood of HIV-Exposed, Seronegative Female Sex Workers in Kenya

**DOI:** 10.1371/journal.pone.0043670

**Published:** 2012-08-22

**Authors:** Duncan Chege, Yijie Chai, Sanja Huibner, Taylor Kain, Charles Wachihi, Makubo Kimani, Samson Barasa, Lyle R. McKinnon, Festus K. Muriuki, Anthony Kariri, Walter Jaoko, Omu Anzala, Joshua Kimani, T. Blake Ball, Francis A. Plummer, Rupert Kaul

**Affiliations:** 1 Departments of Medicine/University Health Network, University of Toronto, Toronto, Ontario, Canada; 2 Department of Medical Microbiology, University of Nairobi, Nairobi, Kenya; 3 Departments of Immunology and Medical Microbiology, University of Manitoba, Winnipeg, Canada; 4 Laboratory for HIV Immunology, National HIV and Retrovirology Laboratories, Public Health Agency of Canada, Winnipeg, Canada; University of California San Francisco, United States of America

## Abstract

**Background:**

Identifying the immune correlates of reduced susceptibility to HIV remains a key goal for the HIV vaccine field, and individuals who are HIV-exposed, seronegative (HESN) may offer important clues. Reduced systemic immune activation has been described in HESN individuals. Conversely, pro-inflammatory T cell subsets, particularly CD4+ T cells producing the cytokine IL17 (Th17 cells), may represent a highly susceptible target for HIV infection after sexual exposure. Therefore, we characterized the cellular pro-inflammatory and IL17/IL22 cytokine immune milieu in the genital mucosa and blood of HESN female sex workers (FSWs).

**Methods and Results:**

Blinded lab personnel characterized basal and mitogen-induced gene and cytokine immune responses in the cervix and blood of HESN FSWs (n = 116) and non-FSW controls (n = 17) using qPCR and ELISA. IL17 and IL22 production was significantly reduced in both the cervix and blood of HESNs, both in resting cells and after mitogen stimulation. In addition, HESN participants demonstrated blunted production of both pro-inflammatory cytokines and β-chemokines.

**Discussion and Conclusions:**

We conclude that HIV exposure without infection was associated with blunted IL17/IL22 and pro-inflammatory responses, both systemically and at the site of mucosal HIV exposure. It will be important for further studies to examine the causal nature of the association and to define the cell subsets responsible for these differences.

## Introduction

Human immunodeficiency virus (HIV) currently infects over 33 million people globally [1], and women are disproportionally affected [1], [2]. While most infected women acquire HIV across the cervical or vaginal mucosa during sex [2], HIV transmission is surprisingly inefficient with an overall risk per coital act of much less than 1% [3]. Nonetheless, there is heterogeneity in susceptibility, and the immune milieu of the female genital tract may be an important determinant of exposure outcome [4]. Individuals who have been HIV exposed but remain seronegative (HESN) have been described in multiple settings and include female sex workers (FSWs) from HIV endemic countries, partners within HIV serodiscordant couples and some men who have sex with men [5]. Understanding the immune correlates of HIV protection in HESN individuals may inform protective vaccine and microbicide design.

**Table 1:** Primer sequences for SYBR green real-time PCR.

mRNA Target	Forward Primer Sequences 5′→3′	Reverse Primer Sequences 3′→5′
**β-Actin**	TCCCTTGCCATCCTAAAAGCCACCC	CTGGGCCATTCTCCTTAGAGAGAAG
**GAPDH**	TGGACCTGACCTGCCGTCTA	CCCTGTTGCTGTAGCCAAATTC
**IFNγ**	AGGGAAGCGAAAAAGGAGTCA	GGACAACCATTACTGGGATGCT
**IL6**	CTGTCCACTGGGCACAGAACT	AAAATAATTAAAATAGTGTCCTAACGCT
**IL17**	CATGAACTCTGTCCCCATCC	CCCACGGACACCAGTATCTT
**IL22**	TGCATTTGACCAGAGCAAAG	AGTTTGGCTTCCCATCTTCC

Glyceraldehyde-3-phosphate dehydrogenase (GAPDH); Interferon Gamma (IFNγ); Interleukin 6 (IL6); Interleukin 17a (IL17); Interleukin 22 (IL22).

Previous studies have begun to elucidate the immune correlates of protection against HIV in HESN individuals. Some individuals manifest virus-specific CD4+ and CD8+ T cell responses, although it is not clear whether these responses are causally protective (reviewed in [6]). More recently, HESN individuals have been shown to demonstrate reduced basal T cell activation in the blood compartment [7], [8], [9], [10] while dampened immune activation *in vivo* has been correlated with reduced cellular susceptibility to HIV infection in culture [9]. Since HIV preferentially replicates in activated CD4+ lymphocytes [11], this relative immune quiescence may decrease the mucosal availability of activated CD4+ T cell targets, making it less likely that sexual HIV exposure will result in productive infection [7], [10], [12]. Indeed, macaque models show that blocking early mucosal inflammation and the recruitment of activated target cells to the genital mucosa was associated with protection from repeated SIV challenge *in vivo* [13]. These findings suggest that a ‘critical mass’ of activated HIV target cells and/or inflammation at the genital mucosa may be required for productive HIV infection [14]. However, it is not known whether immune quiescence extends to genital mucosa, the site of initial HIV-target cell interaction.

**Table 2:** Summary of enrolled study participants.

*Variables*	HESN* sex workers	*Low-risk controls*	*p*
**Demographic variables**			
Age (range)	39 (23–64)	43 (32–51)	0.227
Menopause (%)	9/101 (8%)	2/16 (11%)	0.681
**Clinical variables**			
Genital infections/abnormalities:			
Genital herpes (%)	80/103 (78%)	12/17 (70%)	0.313
Syphilis (%)	3/104 (3%)	0/7 (0%)	0.36
Trichomonas (%)	0/114 (0%)	0/17 (0%)	1
Bacterial vaginosis (%)	21/116 (18%)	1/17 (6%)	0.181
Candidiasis infection (%)	27/116 (23%)	3/17 (18%)	0.531

**Table 3:** Summary of condom use in enrolled female sex workers.

*Self-reported condom use*	*Casual clients* *	*Repeat clients* *	*Regular client/boyfriend* *
**Never (0%)**	0/100 (0%)	0/88 (0%)	46/67 (68%)
**Some (<50%)**	1/111 (<1%)	1/88 (<1%)	2/67 (3%)
**Most (>50%)**	1/111 (<1%)	1/88 (<1%)	5/67 (8%)
**Always (100%)**	98/111 (98%)	86/88 (98%)	14/67 (21%)

* Data was not available for all 116 enrolled sex workers. Respondent numbers are shown for each variable.

The specific CD4+ T cell subsets present at the mucosal site of HIV exposure may also play a role in susceptibility [15]. IL17 producing CD4+ T cells (Th17) cells are important in coordinating mucosal anti-microbial immune responses [16]. However, many [17], [18], [19] (but not all [20]) studies have found that Th17 cells may be preferentially infected by HIV, perhaps because they are activated and terminally differentiated cells that express high levels of CCR5 and α4β7 [19], [21], [22]. IL22 producing CD4+ T cells (Th22 cells) are involved in the maintenance of mucosal integrity and may assist Th17 cells in maintaining antimicrobial immune function [23], but also express high levels of HIV co-receptors and may be preferentially depleted from mucosal sites during HIV infection [19], [24].

It is possible that mucosal HIV acquisition after sexual exposure may be enhanced by an increased number/proportion of mucosal Th17 and Th22 cells and/or mucosal T cell immune activation [12], [18], [19], [24]. Conversely, their decrease may serve as a correlate of immune protection against HIV infection. To evaluate this hypothesis, we examined the genital mucosal immune responses in cervical and blood mononuclear cells within a HESN FSWs cohort from Nairobi, Kenya. Here, we report that HESN FSWs have decreased functional pro-inflammatory, IL17 and IL22 cellular immune responses in the blood, compared to lower risk women; and for the first time extend this functional immune quiescence to the genital mucosa.

**Figure 1:**
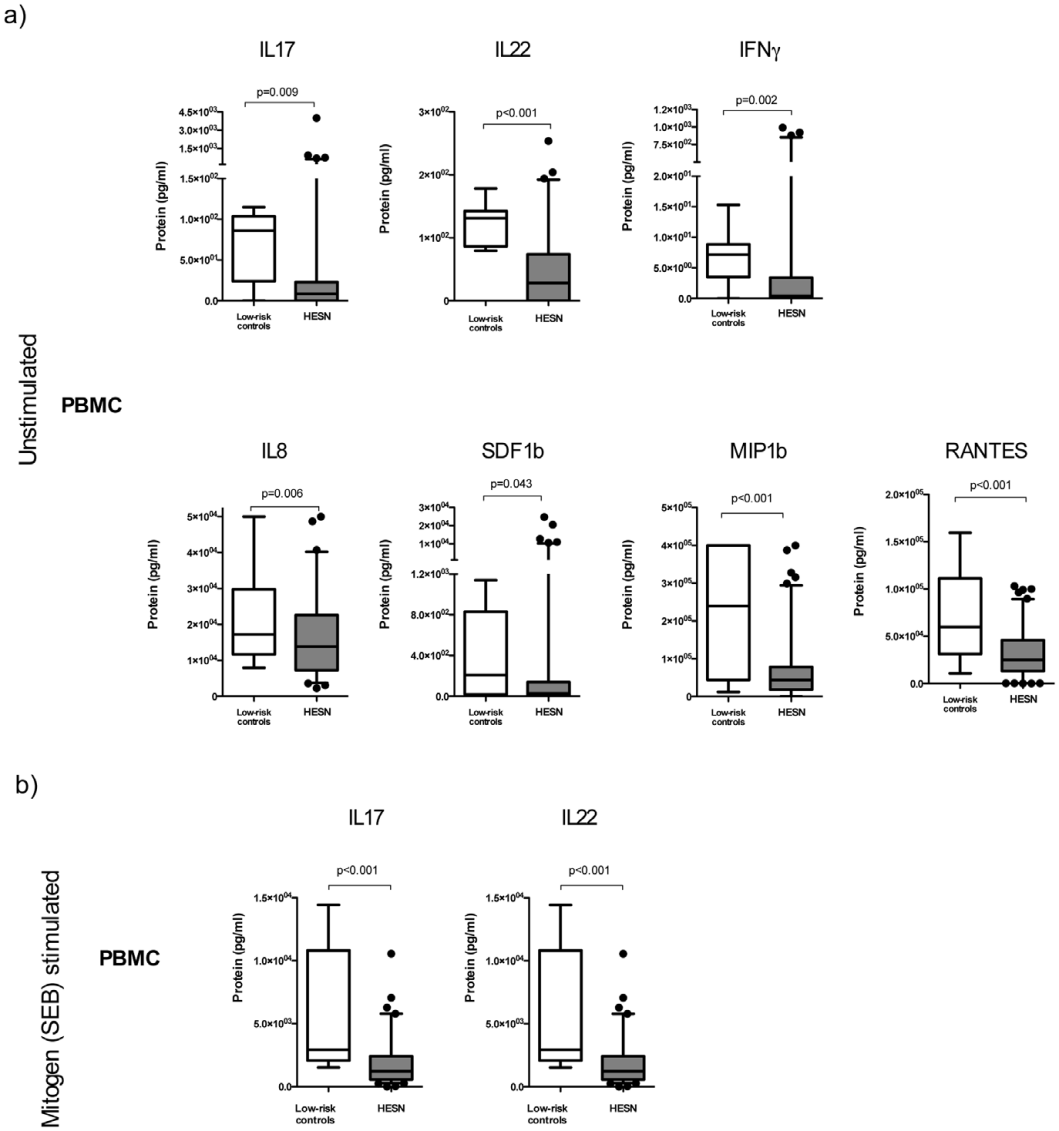
Decreased Th17 and pro-inflammatory cytokine production in HESN blood lymphocytes. Figure shows cytokine levels in supernatants of blood lymphocytes from study participants blood mononuclear cells (a) incubated in culture medium alone (unstimulated), and (b) after SEB mitogen stimulation. Cytokine concentrations (pg/ml; IL17, IL22, IFNg, IL8, SDF-1b, MIP1b, RANTES, IL10, MCP-1, MIP1a, and IL6) in culture supernatants were determined using an ELISA platform, and significant cytokine differences between groups are illustrated. Box and whisker plots are plotted with whiskers covering the 95-5 percentiles, dots representing outliers and horizontal lines representing the median.

**Table 4:** Summary of cytokine production by blood lymphocytes from enrolled participants.

Immune readout (ELISA)	HESN cells inR10 media alone(median)	LRC cells inR10 media alone(median)	P	HESN cells afterSEB stimulation(median)	LRC cells afterSEB stimulation(median)	P
IL6 (pg/ml)	1.386×104	1.722×104	0.234	5.112×103	2.227×103	0.174
IL8 (pg/ml)	5.768×103	6.853×102	**0.006**	6.222×104	1.143×105	0.717
IL10 (pg/ml)	9.860×101	1.037×102	0.932	6.518×102	9.017×102	0.767
IL17 (pg/ml)	8.600×10	8.610×101	**0.009**	1.235×103	2.942×103	<**0.001**
IL22 (pg/ml)	2.800×101	1.312×102	<**0.001**	1.108×103	2.459×103	<**0.001**
IFNg (pg/ml)	4.000×10-1	7.200×10	**0.002**	1.491×103	1.502×103	0.899
SDF-1b (pg/ml)	2.780×101	2.091×102	**0.043**	3.685×101	6.859×102	0.052
MIP-1a (pg/ml)	3.653×104	5.434×104	0.246	2.155×104	1.735×104	0.868
MIP-1b (pg/ml)	4.425×104	2.394×105	<**0.001**	2.435×104	1.019×105	**0.031**
RANTES (pg/ml)	2.514×104	5.994×104	<**0.001**	<1	3.764×102	0.631
MCP-1 (pg/ml)	1.076×104	8.763×103	0.221	1.076×104	1.536×104	0.164

A Mann-Whitney U test was performed to compare differences between groups and the ‘p’ statistic reported. Significant p values (p<0.05) are shown in bold. Staphylococcus enterotoxin B (SEB); HIV exposed seronegative (HESN), Low risk control (LRC); Enzyme linked immunoabsorbent assay (ELISA).

## Methods

### Ethics Statement

All participants provided written informed consent, and study protocols were reviewed and approved by the Research Ethics Boards at the Kenyatta National Hospital, Kenya and the Universities of Manitoba and Toronto, Canada.

### Study Population and Diagnostics

HIV negative FSWs were enrolled from a dedicated sex worker cohort in the Majengo Nairobi area of Kenya. Non-pregnant women with no history of commercial sex work (low-risk controls; LRC), were recruited from the nearby Pumwani Mother-Child Maternity Clinic, where their children obtained routine care. All women were confirmed to be HIV-uninfected using both serology and an optimized sensitive PCR assay specific for *env, nef* and *vif* HIV-1 provirus genes adapted to detect African clades [25], [26]. Herpes simplex type 2 serology was performed using the Kalon IgG enzyme linked immunosorbent assay (ELISA) (Kalon Biological). *T vaginalis* culture was performed (In Pouch TV (Biomed Diagnostics), a gram stain was performed, and blood was tested for syphilis serology (rapid plasma reagin; RPR). Bacterial vaginosis was defined as a Nugent score of 7–10 on the Gram stain [27], and candidiasis was defined as the presence of fungal hyphae. No diagnostics were performed for *N. gonorrhoeae* or *C. trachomatis.* Blood samples and cervical cytobrush specimens were obtained from women who were not actively menstruating, and a behavioural questionnaire was completed.

**Figure 2:**
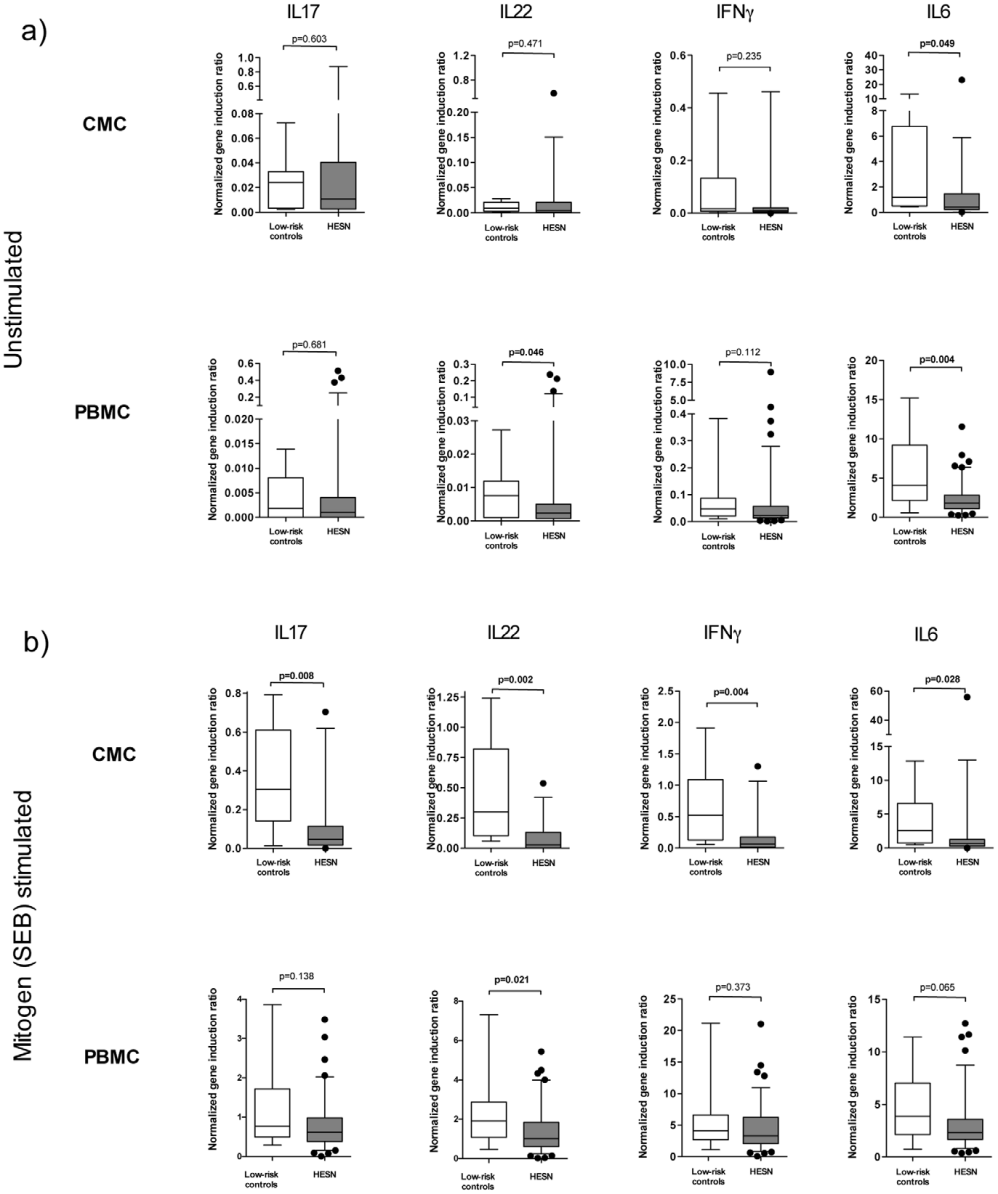
Decreased Th17 and pro-inflammatory cytokine gene expression in the HESN cervix and blood. Figure shows immune gene expression in mononuclear cells from both blood (PBMC) and cervix (CMC) in study participants that were either (a) incubated in culture medium alone (unstimulated), or (b) after SEB mitogen stimulation. A qPCR assay was used to determine mRNA induction of IL17, IL22, IFNg, and IL6 genes. Box and whisker plots with whiskers covering the 95-5 percentiles, with horizontal lines representing the median.

**Table 5:** Summary of immune gene induction in cervical cells from enrolled participants.

Immune readout (qPCR)	HESN cells inR10 media alone(median)	LRC cells inR10 media alone(median)	P	HESN cells afterSEB stimulation(median)	LRC cells afterSEB stimulation(median)	P
**IL17 (ratio)**	0.011	0.024	0.603	0.047	0.304	**0.008**
**IL22 (ratio)**	0.005	0.009	0.471	0.025	0.3	**0.002**
**IFNg (ratio)**	0.01	0.016	0.235	0.065	0.524	**0.004**
**IL6 (ratio)**	0.404	1.189	**0.049**	0.692	2.58	**0.028**

A Mann-Whitney U test was performed to compare differences between groups and the ‘p’ statistic reported. Significant p values (p<0.05) are shown in bold. qPCR data is presented as a ratio of target gene expression over the housekeeping gene. Staphylococcus enterotoxin B (SEB); HIV exposed seronegative (HESN), Low risk control (LRC), qPCR (real time PCR).

### Specimen Collection and *in vitro* Stimulation

Specimen collection and processing was performed as previously described [28], [29]. Briefly, cervical samples were obtained by first scraping the external cervical os using a plastic cell scraper (Benzi Jinshuo Applicator Co) and a cervical histobrush (Histobrush Spectrum Lab) was then inserted into the cervical os and rotated through 360°. A mini-cervical lavage was then performed using 1 ml PBS to collect loosened cells. Then the scraper, swab and mini-lavage cervical samples were combined into a 50 ml sample collection tube containing 10 mls of PBS and transported to the laboratory on ice within 3 hours. Blood was collected by venipuncture into heparin Vacutainer tubes (BD Bioscience). Peripheral blood mononuclear cells (PBMC) and cervical mononuclear cells (CMCs) were isolated by density gradient centrifugation and resuspended in RPMI 1640 media with 10% heat inactivated fetal bovine serum, 1% Penicillin and 1% streptomycin (R10; FBS; Sigma). CMC and PBMC enumeration and viability were assessed by trypan blue exclusion.

**Table 6:** Summary of immune gene induction in blood lymphocytes from enrolled participants.

Immune readout (qPCR)	HESN cells inR10 media alone(median)	LRC cells inR10 media alone(median)	P	HESN cells afterSEB stimulation(median)	LRC cells afterSEB stimulation(median)	P
**IL17 (ratio)**	0.001	0.002	0.681	0.623	0.776	0.138
**IL22 (ratio)**	0.002	0.008	**0.046**	1.017	1.922	**0.021**
**IFNg (ratio)**	0.022	0.039	0.112	3.302	3.964	0.373
**IL6 (ratio)**	1.789	4.077	**0.004**	2.312	3.692	0.065

A Mann-Whitney U test was performed to compare differences between groups and the ‘p’ statistic reported. Significant p values (p<0.05) are shown in bold. qPCR data is presented as a ratio of target gene expression over the housekeeping gene. Staphylococcus enterotoxin B (SEB); HIV exposed seronegative (HESN), Low risk control (LRC), qPCR (real time PCR).

PBMCs (1×10^6^) or all available CMCs were incubated for 6 hours left in culture R10 medium alone (negative control) or stimulated with staphylococcal enterotoxin B superantigen (SEB; Sigma) at 3.0 ug/ml at 37°C in 5% CO_2_. SEB was used as a positive mitogen control to induce IL17, IL22 and other pro-inflammatory cytokine expression and production, similar to other studies [30], [31], [32], [33]. A 6-hour incubation was selected to maximize both protein and RNA yield for targeted immune outputs [34]. After incubation, cell pellets and supernatants were harvested by centrifugation at 10,000 rpm for 5 minutes and cell pellets placed in RNALater solution (Ambion). Both cell pellets and culture supernatants were cryopreserved at −80°C, and were later thawed for quantification of mRNA and protein.

### qPCR and mRNA Quantification

An optimized quantitative real-time PCR (qPCR) assay was used to quantify mRNA as previously described [34]. Briefly, RNA was extracted from cell pellets and genomic DNA was eliminated using the Qiagen RNAeasy Plus Kit (Qiagen) as per manufacturer’s instructions. Complementary DNA (cDNA) was created by adding 10 ng of RNA to the reverse transcription master mix and reverse-transcribed using the Superscript III Kit (Invitrogen) as per manufacturer’s instructions. Genomic DNA (gDNA) standards were isolated from purified placental tissue and used as a universal standard over a 7−log (36 ng –0.4 ng) dilution range to calculate relative gene expression levels [34], [35]. Single intra-exon gene-specific primers were generated using Primer Express Software ™ (Perkin Elmer Applied Biosystems) or OligoPerfect ™ (Invitrogen). Due to recovery of a limited amount of lymphocytes, within cervical cytobrush samples [34], we limited the amount of genes examined via our qPCR assay to only IL17a (IL17), IL22, IL6, and IFNγ mRNA; a list of primer sequences used to detect immune and housekeeping gene mRNA transcription are listed in Table 1.

SYBR green fluorescent dye was used to detect amplification under the following amplification conditions: i) 1 warm-up cycle for 2 min at 50°C ii) 1 pre-amplification cycle for 10 minutes at 95°C, 40 amplification cycles for 15 seconds at 95°C and for 1 minute at 60°C, iii) end-amplification cycle for 15 seconds at 95°C, 15 seconds at 60°C and 15 seconds at 95°C. All reactions were run in triplicate. Quantitative PCR values crossing threshold (Ct) were obtained during the exponential amplification phase using SDS 2.3 Software (Applied Biosystems) and analysed for respective gene quantities and exported into Microsoft Excel for further analysis. Prior validated housekeeping genes, GAPDH and β-Actin [34], were used as the respective reference genes to assess target gene expression in CMC and PBMCs respectively. Gene quantities were calculated from standard curves in arbitrary units; and to assess antigen-specific gene induction, results for each target gene were normalized by dividing the amount of the amplified gene target by the amount of the housekeeping gene for each sample and reported as the normalized gene induction ratio. Since qPCR lacks sensitivity in the context of low CMC counts [34], this assay was only performed in samples having more than 2×10^5^ CMC counts as enumerated using trypan blue exclusion.

### Cytokine Quantification

Cytokine concentrations (pg/ml) in culture supernatants were determined using Searchlight™ chemiluminescent Multiplex-ELISA assay (M-ELISA; Aushon Biosystems, IL17a (IL17), IL6, IL10, MCP-1, IFNγ, RANTES, MIP1α, MIP1β, SDF-1β, IL8) or using a stand-alone ELISA (Platinum™ ELISA, e-Biosciences, IL22) as per manufacturer’s instructions. ELISA assays required relatively little sample volume input and were multiplexed by design, which afforded us the ability to examine a relatively broader range of immune parameters using this output. All samples were run in duplicate. Unknown values were analysed using the 4PL standard curve fit model through Searchlight Array Analyst software ™ (Aushon Biosystems). Cytokine levels in the media control (background) from each participant were subtracted from all presented HIV pool and SEB specific cytokine levels. In our ELISA standard curves, the following criterion was used to report all unknown value data; software reported readings falling below the lowest standard were reported as is, and if reported as ‘undetectable’, a ‘zero’ value was inputted, while no extrapolated values were reported beyond the top known standard. For data analysis purposes, values exceeding the upper standard were reported as the upper limit of detection plus an arbitrary 1 pg/ml.

### Statistical Analysis

Non-parametric Mann-Whitney U, Pearson χ^2^ test and Wilcoxon Signed Ranks statistical analyses were performed using SPSS Version 18.0 software (SPSS Inc.). The number of unprotected sexual HIV exposures over the past year was estimated based on the number and reported condom use with each client group (casual clients, repeat clients and regular partners), assuming an HIV prevalence in male clients of 20% and a condom failure rate of 2%.

## Results

### Study Participants

A total of 116 HIV-uninfected FSWs (HESN FSWs) were recruited from a dedicated female sex worker clinic in Majengo, Nairobi, and 17 lower risk women with no prior history of commercial sex work were recruited from a paediatrics clinic affiliated with the Pumwani Maternity Hospital, Nairobi (low-risk controls; LRC). Age, menopause and the prevalence of genital infections were similar between controls and HESN FSWs (Table 2). Median cervical mononuclear cell (CMC) number was 1.2×10^5^ (range 0–4.38×10^6^) in the HESN FSW group and 2.5×10^5^ (range 0–2.22×10^6^) in the control group. FSW participants had been engaged in commercial sex work for a median of 9 years (range 1–38), and reported a median of 21 casual clients per week (range 3–160). Self reported condom use by FSWs was high with casual clients (98%) and repeat clients (98%), but much lower with boyfriends/regular partners (21%; Table 3). FSW had a median of 30 (range 4–168) estimated unprotected sexual HIV exposures over the past year. The HIV status of FSW male clients was not available.

### Cytokine Production by Blood Lymphocytes in HESN Female Sex Workers

In unstimulated (media incubated) PBMC, the most striking difference between HESN and controls was a reduced production of IL17 (p = 0.009) and IL22 (p<0.001) in the HESN group (Figure 1a). In addition, HESNs also manifested a substantial and consistent reduction in the basal production of pro-inflammatory cytokines, including IFNγ (p = 0.002), IL8 (p = 0.006), SDF-1b (p = 0.043), MIP-1b (p<0.001) and RANTES (p<0.001) (Figure 1a; Table 4). After short-term incubation with the mitogen SEB, HESN participants still demonstrated a much lower production of the cytokines IL17 and IL22 in blood lymphocytes (IL17, p<0.001; IL22, <0.001), Figure 1b), but no difference was now apparent for other pro-inflammatory cytokines or chemokines (Table 4). Overall, the production of IL17 and IL22 was substantially reduced in HESN FSW, both in unstimulated PBMC and after incubation with the mitogen SEB, and basal production of other pro-inflammatory cytokines was also blunted. There was no association between cytokine responses in blood and either the duration of prior sex work, the current number of weekly clients or the calculated number of HIV exposures over the past year (data not shown).

Since HIV acquisition generally occurs in high-risk FSWs after sexual exposure at the level of the genital mucosa, similar assays were attempted using cervical mononuclear cells. However, cytokine and chemokine protein levels in CMC supernatants consistently fell below the level of ELISA detection (∼1 pg/ml), likely due to both the low recovery of mucosal cell numbers obtained from cervical cytobrush sampling [34], as well as the relatively low enrichment of mononuclear lymphocytes within such samples [36].

### Immune Gene Expression in the Blood and Cervix of HESN Female Sex Workers

Generally, qPCR assays offer high sensitivity and in theory could amplify low signals resultant from low cellular input to levels sufficient for relative quantitation [37], [38]. Therefore, we also used a qPCR platform to examine Th17/Th22 associated cytokine responses in the cervix (IL17 and IL22), as well as to confirm our prior ELISA findings regarding reduced pro-inflammatory cytokine production in blood lymphocytes (two cytokines; IL6, IFNγ). In unstimulated CMC there was no difference in IL17 or IL22 gene expression between HESN and control women (Table 5), although IL6 expression was slightly reduced in the former group (p = 0.049; Figure 2a). However, after incubation with the SEB, HESN cervical cells demonstrated substantial and consistently reduced induction of IL17 (p = 0.008) and IL22 (p = 0.002), as well as of the other pro-inflammatory genes (both IFNγ and IL6; each p≤0.028; Figure 2b; Table 5).

Gene expression in the blood followed a similar pattern, with significantly reduced expression of IL22 (p = 0.046) and IL6 (p = 0.004) in unstimulated PBMC from HESN FSWs, but no difference in IL17 (p = 0.681) expression (Figure 2a; Table 6). Following SEB (mitogen) stimulation there was again a relatively reduced expression of IL22 in HESN PBMC (p = 0.021), and a weak trend to reduced IL17 expression (p = 0.138; Figure 2b; Table 6). No association was seen between cervical immune gene responses and either the duration of prior sex work, the number of weekly clients or the calculated number of unprotected HIV sexual exposures over the past year (data not shown). Overall, the qPCR platform confirmed the initial ELISA results, and for the first time expanded these observations to demonstrate blunted IL22 responses and a weak trend towards reduced IL17 production in the cervix of HESN FSWs. In contrast to blood, the reduction in cervical cytokine responses was most apparent in HESNs following mitogen stimulation (rather than in resting cells).

## Discussion

Defining the immune correlates of reduced HIV susceptibility is a key goal for the HIV vaccine field. In this relatively large cohort study, HESN FSWs demonstrated a substantially reduced production of pro-inflammatory and Th17/Th22-type cytokines in both the genital tract and blood compartments, with the blunting of these immune responses most apparent in the cervix after mitogen stimulation. While previous reports have described reduced immune activation in unstimulated blood lymphocytes from HESN individuals [7], [8], [9], [10], [39] and more recently in cervical secretions [40], our current study extends these observations in two important ways. First, we broaden the range of immune responses involved in the ‘immune quiescence’ model of HIV immune protection to include the induction of the classical Th17/Th22 cytokines. Secondly, we are the first to demonstrate that functional immune responses are also quiescent in the genital mucosa, which is the putative site of most HIV acquisition in female sex workers. This is important since increased levels of genital pro-inflammatory cytokines [41] and innate immune factors [42] have been associated with increased HIV acquisition in women from sub-Saharan Africa.

The reduced production/induction of IL17 and IL22 in both the blood and genital tract of HESN participants might be causally related to HIV exposure without infection, since both Th17 and Th22 cell subsets are enriched at mucosal sites and may be particularly HIV susceptible [12], [19], [43], [44], [45] (although not all studies have found this [20]). Th17 (CCR4+CCR6+) cells have been shown to harbour higher HIV proviral levels than other CD4 subsets *ex vivo* [46], and blocking of MIP3a, the natural CCR6 ligand, can protect macaques from repeated SIV challenge *in situ* [13]. Additionally, Th17 cells express elevated levels of the surface marker α4β7 and preferentially bind HIV gp120 envelope [19], express increased levels of the HIV entry co-receptor CCR5 [18], [19], and produce lower levels of CCR5 binding β-chemokines [18] potentially increasing their susceptibility to HIV infection. Interestingly, the pro-inflammatory cytokine IL6 is a critical cofactor in the development of Th17 cells [47], and was also reduced in both the blood and genital tract of HESN participants.

In addition to relative immune quiescence, HESN status has been associated with HIV-specific T cells with a pro-inflammatory functional profile (reviewed in [6]). This seems at odds with the findings of immune quiescence in our study, as well as in others [7], [8], [9], [10], [40]. While these two observations appear paradoxical, this need not be the case [7], [10]. Cellular immune quiescence in the genital mucosa may result in a reduced pool of susceptible CD4+ T cells after initial sexual HIV exposure, reducing HIV acquisition risk, and repeated exposure may then generate the mucosal HIV-specific T cell responses that have been observed. Whether these responses are a paraphenomenon of exposure or contribute to protection is not known [48], [49], [50], but their low frequency may mean that they can contribute to protection without greatly increasing the number of mucosal activated cells [7], [10], [51]. Therefore, these two immune correlates may work in tandem to protect against HIV.

Despite these interesting results, it is important to acknowledge potential limitations to our study. Our immune assays do not permit identification of the exact cell subpopulations responsible for measured immune responses. While IL17 and IL22 fall under the broad category of “Th17” type cytokines, several cell types other than classical CD4+ αβ T cells that are isolated by density centrifugation can also produce them, including γδ T cells, and NKT cells [52]. Future cytometric studies will be needed to further identify these cells. Additionally, although we were readily able to measure gene induction in the genital mucosa, the inability to measure protein production here using a sensitive ELISA platform might suggest that these cells could be anergic and this would need to be evaluated in further immunologic studies. While the two platforms used (qPCR and ELISA) were generally in agreement, subtle differences in results were apparent. Discordant kinetics of mRNA expression and protein production levels may explain this observation. Peak mRNA levels and their subsequent decline may have preceded the peak protein production levels attained at the end of the 6-hour incubation period [34]. Alternatively, transcript and protein concordance has been reported to vary within various settings, and could correlate anywhere from 17%–100% [53], [54], [55] such that measured mRNA levels may only be partially indicative of protein secretion. Nevertheless, despite some inter-platform variability, our results consistently show that HESN FSWs have an immuno-quiescent phenotype in blood (ELISA and qPCR) and cervix (qPCR), expanding several other studies identifying immune quiescence in blood as a correlate of relative HIV protection [7], [8], [9], [10], [39], as well as a distinct pattern of soluble immune factors in genital secretions [40].

We estimated the number of unprotected HIV exposures over the past year, based on current condom use and numbers within various client groups, and assuming that the HIV prevalence in all male partners was 20%. However, our immune studies did not find any association between this calculated annual HIV exposure rate, or other sex work parameters with the magnitude of immune responses in sex workers. Some studies have defined relative HIV ‘resistance’ in female sex workers based on criteria such as a threshold duration of prior follow up [26]. However, in this study we defined all HIV-uninfected sex workers as potentially HIV-exposed but seronegative (HESN), since all women had been engaged in commercial sex work for at least one year prior to cohort enrolment. The HIV prevention program that is offered to all cohort participants results in a very rapid increase in condom use and reduction in client numbers after enrolment [56], so that past HIV exposure may have been considerable despite low current sexual risk. In addition, despite high condom use with casual clients, condoms are infrequently used with regular partners and this represents a poorly-understood source for ongoing HIV exposure. These potential confounders mean that it is difficult to precisely correlate immune parameters with a quantitative measure of past HIV infection pressure.

The cross-sectional design of our study means that we cannot prove that the association between the HESN phenotype and immune quiescence is causal. Our eventual goal is to address this issue using prospective studies with HIV acquisition as an outcome. Frequent sexual activity amongst sex workers might conceivably confound our genital immunology results due to repeated exposure to semen, known to have both immunoregulatory and pro-inflammatory effects [48]. However, since immune quiescence was also found in blood, it is unlikely that frequent cervical semen exposure explains the immune differences seen in HESN sex workers. Finally, Herpes simplex type 2 (HSV-2) infection is associated with genital mucosal inflammation and recruitment of T cell and innate immune cells [29], [57]. Most participants in the study were HSV-2 infected, and screening for asymptomatic reactivation was not performed. However, since HSV-2 was more common in the HESN women, and prior studies have shown that the pro-inflammatory mucosal immune impact of HSV-2 in HESN women is similar to controls [58], this is not likely to lead to our finding of HESN immuno-quiescence.

In summary, we find that HESN female sex workers demonstrated substantial and consistent reduction in the basal and mitogen-stimulated production of Th17/Th22-associated and pro-inflammatory cytokines in both the genital and blood compartments. These immune correlates of reduced HIV susceptibility may provide important clues for future vaccine and microbicide research.
